# Editorial Note: Factors influencing walking trips. Evidence from Gdynia, Poland

**DOI:** 10.1371/journal.pone.0348562

**Published:** 2026-05-05

**Authors:** The PLOS One Editors

The *PLOS One* Editors issue this Editorial Note because this article [1] was identified as one of a series of submissions for which we have concerns about aspects of the peer review process.

The authors stated that they were unaware of the issues, and the Editors have no concerns regarding their contributions to peer review.
